# Mindfulness moderates the relationship between emotional eating and body mass index in a sample of people with cystic fibrosis

**DOI:** 10.1007/s40519-020-00969-6

**Published:** 2020-07-31

**Authors:** Helen Egan, R. Keyte, E. F. Nash, J. Barrett, A. Regan, M. Mantzios

**Affiliations:** 1 Department of Psychology, Faculty of Business, Law and Social Sciences, Birmingham City University, Room C307, The Curzon Building, 4 Cardigan St., Birmingham, B4 7BD UK; 2 University Hospitals Birmingham NHS Foundation Trust, West Midlands Adult Cystic Fibrosis Centre, Birmingham Heartlands Hospital, Ward 26, Bordesley Green East, Birmingham, B9 5SS UK

**Keywords:** Cystic fibrosis, Eating behaviours, Mindful eating, Mindfulness, Self-compassion

## Abstract

**Purpose:**

Self-regulation in eating is significant for enhancing life expectancy of people with cystic fibrosis (CF), but research with this population is scarce.

**Methods:**

In a cross-sectional study, adults with CF completed a number of psychometric scales exploring typical eating behaviours that may increase calorific intake including motivations to eat palatable foods and scales that may be associated with decreased calorific intake: mindfulness, mindful eating and self-compassion.

**Results:**

Findings suggested that motivations to eat palatable foods and eating behaviours correlate with higher BMI, while mindfulness, mindful eating and self-compassion did not reach significance. Mindfulness and mindful eating moderated the relationship between emotional eating and BMI, while self-compassion did not moderate this relationship.

**Conclusions:**

There is a need to develop healthy and effective means of enhancing calorific intake, where this is indicated, adapting mindful eating principles to focus on increasing both self-regulation and pleasure in eating while reducing emotional eating may be one means of doing this.

**Level of evidence:**

Level V, cross-sectional descriptive study.

## Introduction

Cystic Fibrosis (CF) is a common life-limiting genetic disease with increasing numbers across the world [1, 2]. CF gets in the way of producing adequate digestive enzymes, leading to the abnormal digestion and malabsorption of nutrients [3]. CF requires daily “preventative management and symptomatic treatment” [4] with much emphasis on maintaining a healthy body weight by eating a high-energy diet accompanied with pancreatic enzyme replacement therapy and fat soluble vitamins to avoid the development of malnutrition [1]. Maintaining optimal nutritional status and body mass index (BMI) improve the quality of life and prolong life expectancy of adults with CF [5]. Undernourishment is significant to the physiological health of people with CF, and while there are concerns with growing rates of obesity in this population and there is a need to balance the benefits of higher BMI with longer term health risks of being obese, a lower BMI continues to present a significant health risk [6]. Despite adults with CF learning to adopt highly regulated eating environments at an early age, research has not focused on ‘conventional’ eating behaviours such as emotional eating.

Emotional eating is usually described as the reaction of overeating in response to emotions [7], in other words, the regulation of emotions occurs through a temporary, and often repetitive response, to eat and overeat. In weight regulation literature, emotional eating is associated with weight dysregulation through a number of means including binge eating, unnecessary fluctuations, and an inability to reach and maintain the optimal weight range for the individual [8, 9]. While emotional eating is a behavioural construct that has been construed as problematic in contemporary eating behaviours, there is little evidence on this with people with CF, how they eat and interact with their food is largely unknown. Understanding more about emotional eating and other eating behaviours offers the potential for improving eating behaviours and, therefore, wellbeing for this population.

Mindfulness may offer a significant resource for supporting the psychological wellbeing for people with CF. Kabat-Zinn [10] described mindfulness as an awareness that emerges through purposefully paying attention in the present moment, non-judgmentally. Relevant to aiding people with emotional eating, Lattimore [11] proposed a mindfulness-based programme to assist with stripping away emotions from the actual behavior of eating. Grossman et al. [12], found that mindfulness-based stress reduction helped individuals cope with clinical and non-clinical worries, and suggested that mindfulness training could help people cope with chronic disease and stress. Systematic reviews and meta-analyses of randomised controlled trials provided an evidence base suggesting mindfulness practices supported those with depression and anxiety [13, 14]. Other research around mindfulness programmes similarly suggests that there is a plethora of benefits when dealing with long-term conditions. The effectiveness of (a) mindfulness-based stress reduction (MBSR) [15] for stress [16], (b) mindfulness-based cognitive therapy (MBCT) [17, 18],for depression [19] and (c) mindfulness-based eating awareness training (MB-EAT) [20], or mindfulness and self-compassionate interventions [21, 22] for nutritional adherence demonstrates an improvement in psychological and physiological health through the use of mindful practices.

Mantzios et al. [23] proposed that there is much unexplored value to the literature that has attempted to explain the obesity ‘epidemic’, they posited that knowledge from research on distracted or inattentive eating, which disadvantages most people in resisting food, could be utilized to support people with CF to eat the amounts needed to avoid malnourishment. Given the importance of offering psychosocial support alongside physiological care to enhance quality of life and life expectancy in CF, Egan and Mantzios [24] further suggested that there is much potential for eating-specific mindfulness to be used to enhance the pleasure and non-medicalized perceptions of food, and introduce or reinforce a healthy relationship with food. Exploring mindfulness and mindful eating further was considered to be the next step in aiding people with CF to manage eating behaviours while also targeting optimal food intake and weight.

The present research aimed to firstly explore eating attitudes and behaviours, and how they relate to body mass index (BMI) and secondly to explore the moderating effect of mindfulness and mindful eating on eating attitudes and behaviours.

## Methods

### Participants

All English speaking patients in a regional Adult Cystic Fibrosis Centre in the United Kingdom who were attending a routine outpatient appointment, or were receiving care on an inpatient ward on pre-arranged data collection days over a period of 6 months, were invited to take part. Ninety-two adults with cystic fibrosis were recruited through a regional Adult Cystic Fibrosis Centre (UK) during scheduled appointments. The sample (*M*_age_ = 30.80, SD = 10.65; *M*_BMI_ = 23.19, SD = 4.03; females = 45, males = 33, not disclosed = 14) consisted of White British (*n* = 83) and nine participants who did not disclose any ethnic background. None of the participants were smokers, and they were recruited on a voluntary basis, and did not receive any financial rewards.

### Materials

#### Participant information sheet

Participants were asked to report their age, gender, height, weight, ethnicity, and smoking.

#### Self-compassion scale (SCS) [25]

The SCS scale is a 26 item self-report measure. Responses range from 1 (*almost never*) to 5 (*almost always*), with overall scores ranging from 26 to 130. Sample items include ‘I try to be loving towards myself when I’m feeling emotional pain’ (i.e., self-kindness) and ‘When I'm down and out, I remind myself that there are lots of other people in the world feeling like I am’ (i.e., common humanity). The scale is composed of six subscales, with alphas of: self-kindness (*α* = 0.70), self-judgment (*α* = 0.89), common humanity (*α* = 0.72), isolation (*α* = 0.81), mindfulness (*α* = 0.72) and over-identification (*α* = 0.83). The present study produced an overall Cronbach’s alpha of 0.93 for the total score.

#### Five Facet Mindfulness Questionnaire-Short Form (FFMQ-SF) [26]

The FFMQ-SF is a 24-item questionnaire measuring five main characteristics of mindfulness, and is based on the original 39-item version (FFMQ) [27]. Responses range from 1 (*never or rarely true*) to 5 (*very often or always true*), with total scores varying from 24 to 120. Sample items are ‘I find it difficult to stay focused on what’s happening in the present moment’ (i.e., acting with awareness) and ‘usually when I have distressing thoughts or images I can just notice them without reacting’ (i.e., non-reactive), and higher scores indicate higher levels of mindfulness. The five measured facets produced an alpha: observing (*α* = 0.71), describing (*α* = 0.83), acting with awareness (*α* = 0.86), non-judging (*α* = 0.82) and non-reactivity (*α* = 0.74). The present study produced an overall Cronbach’s alpha of 0.87 for the overall score.

#### Mindfulness Eating Scale (MES) [28]

The MES is a 28 item scale, and is combined with five subscales, with responses ranging from 1 (*Never*) to 4 (*Usually*), and overall scores varying from 28 to 112. Sample items include ‘I wish I could control my eating more easily’ (i.e., acceptance) and ‘I notice flavours and textures when I'm eating my food’ (i.e., awareness). Higher scores indicate higher levels of mindful eating. The five subscales produced an alpha of: acceptance (*α* = 0.93), awareness (*α* = 0.82), non-reactivity (*α* = 0.75), routine (0.85), distractibility (*α* = 0.93) and unstructured (0.71). The present study produced an overall Cronbach’s alpha of 0.93 for the total score.

#### Three Factor Eating Questionnaire-Short Form (TFEQ-R18) [29]

The TFEQ-R18 is an 18 item questionnaire and measures the concepts of restrained eating, emotional eating and uncontrolled eating, and is based on the original 51 item–version (*TFEQ)* [30]. It includes items such as ‘When I smell a delicious food, I find it very difficult to keep from eating, even if I have just finished a meal.’ (i.e., restrained eating) and ‘When I feel lonely, I console myself by eating’ (i.e., emotional eating). Responses range from 1 (*definitely false*) to 4 (*definitely true*), with overall scores ranging from 18 to 76. The three subscales produced an alpha of: restrained eating (*α* = 0.77), emotional eating (*α* = 0.86) and uncontrolled eating (*α* = 0.77). The present study produced an overall Cronbach’s alpha of 0.83 for the overall score.

#### The Palatable Eating Motives Scale (PEMS) [31]

The PEMS consists of 19 items which assess motives for eating palatable but unhealthy foods for reasons other than hunger. On a 5-point Likert scale, responses range from 1 (never/almost never) to 5 (always/almost always) and scores range from 19 to 95. A variety of foods are listed (e.g., sweets like ice cream, chocolate, doughnuts, cookies, cake, candy, muffins, scones, fudge, brownies, and other desserts), with instructions stating for participants to think about times they have eaten any of the listed foods, and for them to mark how often they have consumed the foods for the following reasons. Sample items include ‘I consume these foods/drinks to forget my worries’ and ‘I consume these foods/drinks to get “high like” or euphoric feelings’. The PEMS factors into four motives, alpha scores and descriptions for each motive are presented: coping motives (*a* = 0.92) include consuming the listed foods to help deal with negative states (e.g., to help with worry, depression or nervousness), reward enhancement motives (*a* = 0.87) include consuming the palatable foods and beverages to enhance a positive experience or emotion, because it is rewarding (e.g., because it is fun, or feels pleasant), social motives (*a* = 0.93) relate to eating the palatable foods or beverages for social reasons, (e.g., to enjoy a party or to be more sociable) and conformity motives (*a* = 0.85) pertain to eating the foods and drinks because of pressure by others (e.g., to fit in). The present study produced an alpha of (*a* = 0.95) for the PEMS.

### Procedure

Potential participants received a participant information form, consent form, followed by the demographic information page and the questionnaires. Participants either completed the hardcopy questionnaire in hospital or at home. Once participants completed the study, they were directed to a debriefing form, which provided them with further information about the aim and purpose of the current study. Participants were also given the opportunity to record an arbitrary number, which would allow them to withdraw their data at a later stage and retain the anonymity of participation. Ethical approval was granted by the Ethical Committee based within the University and by the National Institute for Health Research (NIHR).

### Analyses

All statistical analyses were conducted using IBM SPSS 24. Data was initially explored through bivariate correlations. Moderation effects were interpreted using PROCESS (Model 1) with a bootstrap sample of 5000, where variables were centered to their means [32]. Simple effects coefficients were computed for three values of the moderator (i.e., 1 SD below the mean, at the mean, and 1 SD above the mean). For all analyses, *p* values ≤ 0.05 were considered statistically significant; nevertheless, the bootstrapping procedure and use of bias-corrected confidence intervals (CI) was determined to attribute statistical significance of the moderator [33]. Partial *η*^2^ was used as the effect size measure in all analyses. Benchmarks for partial *η*^2^ are 0.01, small; 0.06, medium; and 0.14, large.

## Results

Bivariate correlation coefficient (i.e., Pearson’s) and significance values between BMI and variable scores (i.e., mindfulness, self-compassion, mindful eating, motivations to eat palatable foods and the three factor eating subscales), as well as the scale and subscale means and standard deviations are presented in Table 1. Significant positive associations were observed between BMI and motivations to eat palatable foods, emotional eating and cognitive restraint, (marginally non-significant), thus these eating behaviours are associated with higher BMI. Mindful eating, and traits such as mindfulness and self-compassion did not significantly relate to BMI (although mindful eating and mindfulness appeared to have a negative coefficient, while self-compassion did not).

**Table 1:** Means and standard deviations of variables, and bivariate correlations to BMI

Variables	*M*	SD	Correlation to BMI	
			*r*	*p*
Mindfulness	74.18	15.12	− 0.065	0.589
Self-compassion	74.88	18.08	0.002	0.990
Mindful eating	84.15	10.81	− 0.151	0.211
Motives to eat palatable foods	42.70	15.17	0.252	0.036
Cognitive restraint	9.37	3.45	0.212	0.059
Uncontrolled eating	18.52	5.31	0.010	0.931
Emotional eating	5.39	2.55	0.283	0.017

**Fig. 1:**
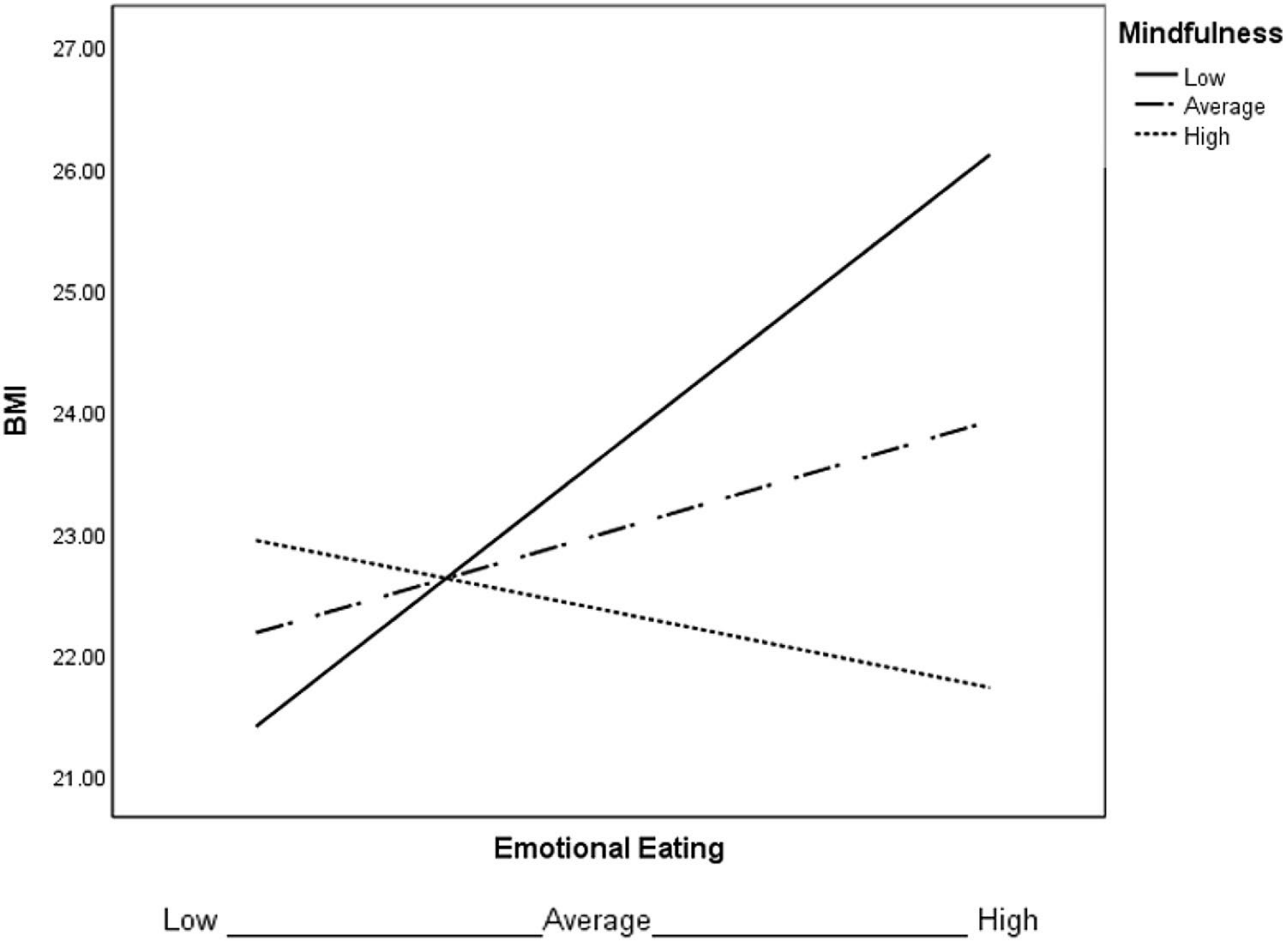
Moderation model of emotional eating predicting BMI for 1 SD below the mean of mindfulness (low), the mean of mindfulness (average), and 1 *SD* above the mean of mindfulness (high)

**Fig. 2:**
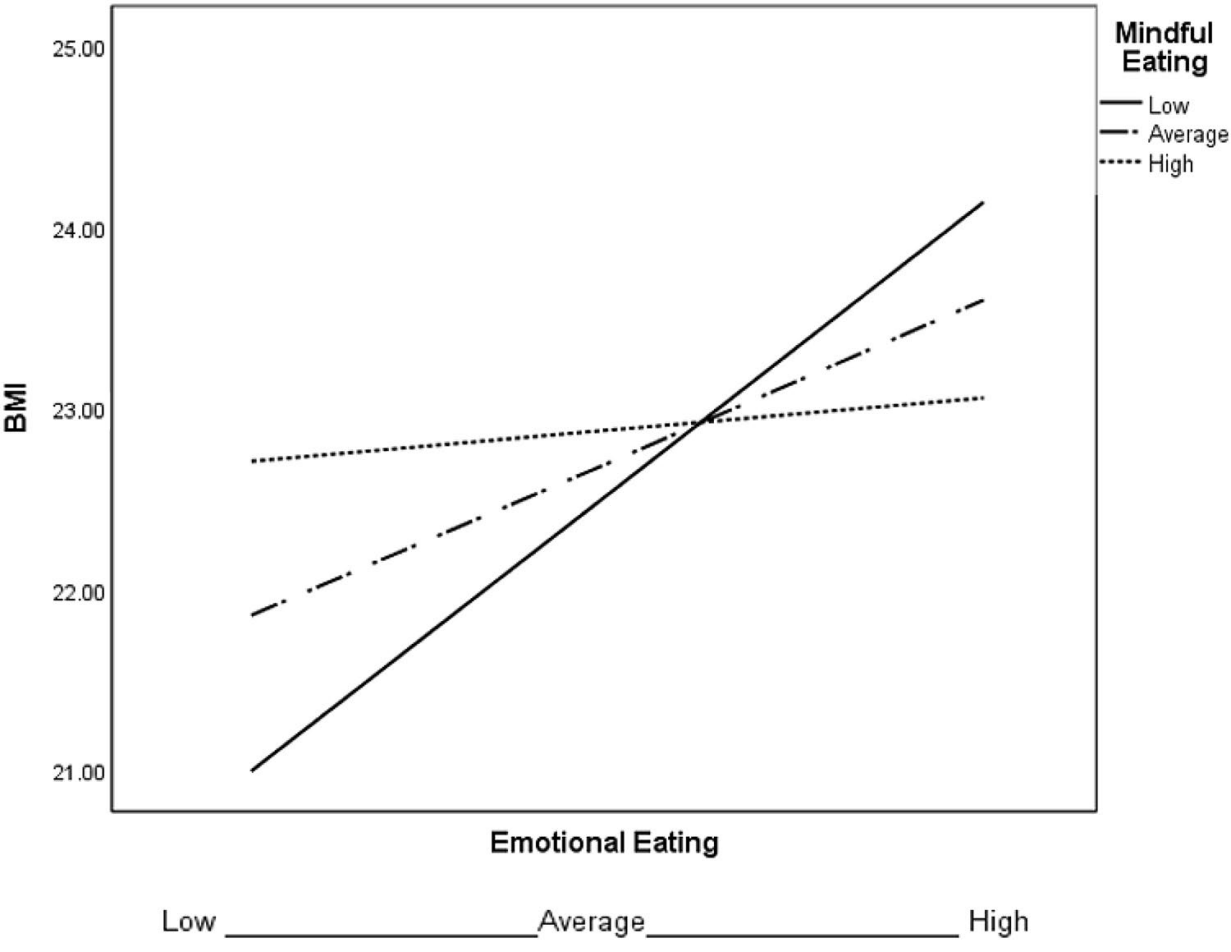
Moderation model of emotional eating predicting BMI for 1 SD below the mean of mindful eating (low), the mean of mindful eating (average), and 1 SD above the mean of mindful eating (high)

Further explorations related to testing mindful eating, mindfulness and self-compassion as potential moderators of observed relationships between BMI and emotional eating. Self-compassion was not a significant moderator, which failed at the highest order interaction (*F*(1, 67) = 1.42, *p* = 0.24, Δ*R*^2^ = 0.02), while mindfulness and mindful eating were significant (see Table 2; Figs. 1, 2, respectively). Results indicate that the significant positive relationship between emotional eating and BMI becomes insignificant as mindfulness or mindful eating scores increase.

**Table 2:** Conditional effects of mindfulness and mindful eating on the relationship between emotional eating and BMI

	*β*	*p*	95% CI	
Mindfulness				
− 1 SD	0.95	< 0.001	0.37	1.54
At the mean	0.35	0.06	− 00.01	0.72
+ 1 SD	− 0.25	0.50	− 0.97	0.47
Mindful eating				
− 1 SD	0.63	< 0.01	0.18	1.09
At the mean	0.35	0.13	− 0.11	0.81
+ 1 SD	0.07	0.82	− 0.54	0.68

*SD* standard deviation, *CI* confidence intervals, *p* significance level, *β* regression coefficient

## Discussion

The aim of this research was to explore eating attitudes and behaviours in relation to BMI and the corresponding moderating role of mindfulness and mindful eating. Findings indicated that for adults with CF, the relationships had some similarities with non-clinical populations. With a primary focus on BMI and ascertaining potential new relationships that are relevant to weight regulation; motivations to eat palatable foods, cognitive restraint, and emotional eating were positively associated with higher BMI, which has been observed in other research [34]. In other words, these eating behaviours are associated with higher BMI for people with CF. Mindfulness, self-compassion and mindful eating did not significantly relate to BMI, but mindful eating and mindfulness appeared to have a negative coefficient, while self-compassion did not, which further corresponds to previous research with non-clinical samples [35, 36]. The differential relationship observed with self-compassion also corresponds to other research and literature in the field of eating and weight regulation [37, 38]. Further explorations showed that mindfulness and mindful eating are moderators of the relationship between emotional eating and BMI. In other words, for adults with CF who score low in mindfulness and/or mindful eating, there is a significant relationship between emotional eating and BMI, while higher scores make this relationship non-significant. Results are consistent with other literature that has explored the associations between emotional eating, BMI and wellbeing, as well as emotional eating, and mindfulness-based interventions [39, 40]. In essence, mindfulness and mindful eating do not only propose a way to relate to food that is centered in the present moment and each meal, but is also relevant in reinforcing emotional regulation, in many ways weakening the presence of strong and distressing emotions, disentangling them from the whole experience of eating [41].

While a higher BMI is often a desirable aim for adults with CF, the potential of using interventions which increase emotional eating to meet this aim are problematic as an increase in levels of emotional eating in addition to other known issues such as pressure from others to eat [42] is not desirable for holistic health and wellbeing, and is associated with other problematic eating behaviours such as binge eating and grazing.

A limitation of this work is that it is cross-sectional in nature, though this was a necessary first step as a method of risk assessment in this clinical population, present findings are, therefore, associative by nature. The sample included was from a large regional unit with a wide geographical base and included all eligible and consenting outpatients on set days; hence, characteristics of participants are likely to be comparable to other CF patients across the UK. Information on previous or current experience with mindfulness practices was not sought with the aim of keeping participants completely blind to the nature of enquiry. Future research should aim to incorporate this information alongside more detailed current treatment regimen information.

Future research should explore current findings further utilizing different mindfulness interventions and practices to enhance the support for people with CF. Such interventions should consider the particular needs of people with CF including shorter practices to accommodate treatment demands, or practices such as colouring which circumvent potential difficulties with deep breathing and/or reduced mobility which may impact on some traditional practices such as yoga,

There is a pressing need is find holistic ways of supporting healthy eating behaviours which take into account the management of co-morbid conditions which impact on eating and also address psychological difficulties and barriers to eating well. Given the complex fluctuating symptoms and extensive burden of treatments for adults with CF this is a considerable challenge. This research proposes the potential of mindfulness and mindful eating to create a focus on self-regulation and increasing pleasure in eating which may create a more balanced approach to eating that results in improved weight and BMI without detriment to mental health and wellbeing that is associated with emotional eating.

- What is already known on this subject?

Cystic fibrosis symptoms impact strongly on eating behaviours and nutritional and weight status which is closely linked to respiratory status, quality of life and life expectancy with malnutrition remaining a significant problem within CF. There is a paucity of research on eating experiences and behaviours on adults with cystic fibrosis, the limited evidence available focuses on eating disorders and disordered eating, and while these are not more prevalent, disturbed eating attitudes and behaviours are evident. There is no evidence around mindful eating within cystic fibrosis and a fuller understanding is needed to align any interventions with good psychological wellbeing.

- What does this study add?

Higher levels of emotional eating were associated with a higher BMI in a sample of people with cystic fibrosis. While a higher BMI is often the aim and is desirable, an increase in levels of emotional eating is not desirable for holistic health and wellbeing. Both mindfulness and mindful eating moderated this relationship. This research proposes the potential of mindfulness and mindful eating in creating interventions to improve physiological outcomes of weight and BMI while also supporting psychological wellbeing.

## Data Availability

The data that support the findings of this study are available on request from the corresponding author. The data are not publicly available due to public availability violating the consent that was given by research participants.
